# Some Like It Hot: The Influence and Implications of Climate Change on Coffee Berry Borer (*Hypothenemus hampei)* and Coffee Production in East Africa

**DOI:** 10.1371/journal.pone.0024528

**Published:** 2011-09-14

**Authors:** Juliana Jaramillo, Eric Muchugu, Fernando E. Vega, Aaron Davis, Christian Borgemeister, Adenirin Chabi-Olaye

**Affiliations:** 1 Institute of Plant Diseases and Plant Protection, University of Hannover, Hannover, Germany; 2 International Center of Insect Physiology and Ecology (icipe), Nairobi, Kenya; 3 Sustainable Perennial Crops Laboratory, United States Department of Agriculture, Agricultural Research Service, Beltsville, Maryland, United States of America; 4 Royal Botanic Gardens, Kew, Richmond, Surrey, United Kingdom; National Institute of Water & Atmospheric Research, New Zealand

## Abstract

The negative effects of climate change are already evident for many of the 25 million coffee farmers across the tropics and the 90 billion dollar (US) coffee industry. The coffee berry borer (*Hypothenemus hampei*), the most important pest of coffee worldwide, has already benefited from the temperature rise in East Africa: increased damage to coffee crops and expansion in its distribution range have been reported. In order to anticipate threats and prioritize management actions for *H. hampei* we present here, maps on future distributions of *H. hampei* in coffee producing areas of East Africa. Using the CLIMEX model we relate present-day insect distributions to current climate and then project the fitted climatic envelopes under future scenarios A2A and B2B (for HADCM3 model). In both scenarios, the situation with *H. hampei* is forecasted to worsen in the current *Coffea arabica* producing areas of Ethiopia, the Ugandan part of the Lake Victoria and Mt. Elgon regions, Mt. Kenya and the Kenyan side of Mt. Elgon, and most of Rwanda and Burundi. The calculated hypothetical number of generations per year of *H. hampei* is predicted to increase in all *C. arabica*-producing areas from five to ten. These outcomes will have serious implications for *C. arabica* production and livelihoods in East Africa. We suggest that the best way to adapt to a rise of temperatures in coffee plantations could be via the introduction of shade trees in sun grown plantations. The aims of this study are to fill knowledge gaps existing in the coffee industry, and to draft an outline for the development of an adaptation strategy package for climate change on coffee production. An abstract in Spanish is provided as Abstract S1.

## Introduction

The Intergovernmental Panel on Climate Change (IPCC) [1] predicts an increase in the mean global temperature of 1.4° to 5.8°C by the end of the twenty-first century [2]. For Africa, future annual warming ranges from 0.2°C (B1 scenario) to >0.5°C per decade (A2 scenario) [1], [3]. Future changes in mean seasonal rainfall in Africa are less well defined. However, in general, models forecast that parts of equatorial East Africa will likely experience 5–20% increase in rainfall from December to February and 5–10% decrease in rainfall from June to August by 2050 [3]. Climate change is also projected to cause more frequent and intense El Niño-Southern Oscillation (ENSO) events leading to widespread drought in some areas and extensive flooding in others [4]. Consequently, such events will have negative impacts on the availability of water resources, food and agricultural security, human health and biodiversity. These changes in climatic conditions are also predicted to profoundly influence the population dynamics and the status of agricultural insect pests [5]–[7] as temperature has a strong and direct influence on insect development, reproduction and survival [7]. Over the past 30 years or so, changing climate and in particular global warming has already produced numerous shifts in the distribution and abundance of species [8]–[9]. Climate change and invasive species are considered as two of the most important ecological issues facing the world today [10].

Coffee (*Coffea arabica* L. and *C. canephora* Pierre ex A. Froehner) is the world's most valuable tropical export crop, with an annual retail value of approx. US $ 90 billion. *Coffea arabica* prices have increased by 160% during the last two years [11]. This is mainly due to production shortages, which, among other reasons, like underproduction which has occurred in several countries as a result of coffee growers reducing the size of their plantations or abandoning them altogether, as a consequence of a long cycle of lowest-ever world market prices caused by over- production and technological change between 2000–2002, is also due to increasing temperatures and consequent damages by pests and diseases [12].

The coffee berry borer, *Hypothenemus hampei* (Ferrari) (Coleoptera: Curculionidae: Scolytinae), is the most important biotic constrain for commercial coffee production worldwide [13], [14]. The geographic centre of origin of the coffee berry borer is unknown, but it is probably endemic to central Africa, becoming naturalized elsewhere due to movement of coffee plants and beans through multiple, persistent introductions [15]. Until ten years ago, there were no reports of *H. hampei* found attacking coffee plantations above 1,500 m, which is within the preferred altitude range of cultivated and naturally occurring *C. arabica* (1,400–1,600 and 1200–2000 m.a.s.l., respectively) [16], suggesting that the original host of the coffee berry borer was probably *C. canephora* [13], [17], [18], a species naturally occurring and cultivated at lower altitudes (250–1500 m.a.s.l.). However, due to recent increasing temperatures in coffee growing regions in the world the insect can now be found also at higher altitudes, where it able to infest *C. arabica* [17]. It is unknown if *C. arabica* and *C. canehora* are the only host plants of *H. hampei*. Other *Coffea* species, or perhaps even other genera of indigenous Rubiaceae, which both occur in large numbers in the understory of forests in Africa, are also attacked by the coffee berry borer under natural conditions. There are many reports of feeding, with occasional reproduction, in plants of the Fabaceae family and reports of three Rubiaceae species where feeding and reproduction of the borer has taken place [13], but no detailed studies on life table parameters of the borer on those plants have been conducted. The coffee berry borer attacks the beans, which are the marketable product, causing losses exceeding US $500 million annually, and worldwide affects many of the more than 25 million rural households involved in coffee production [19]. Under low pest pressure the conversion factor (i.e. after processing, the amount of parchment coffee obtained from a given amount of freshly picked coffee berries) is 5∶1; however, a serious *H. hampei* infestation can alter this ratio up to >17∶1, with devastating economic consequences for farmers [20]. Currently, *H. hampei* is present in all coffee producing areas of the world, except China and Nepal, with the most recent introductions to Puerto Rico in 2007 and Hawaii in 2010.

Earlier predictions on the effects of climate change on coffee and the coffee berry borer estimated that even a small increase in temperature would have serious consequences for coffee production, including plantations in Brazil, Mexico and Uganda, in some cases rendering production very difficult [21]–[23]. Particularly serious consequences are predicted for the areas where high quality *C. arabica* is produced [17]. Jaramillo et al. [17] predicted that a 1°C increase would lead to a considerably faster development, higher number of generations per fruiting season and a shift in the geographical range for *H. hampei*. Furthermore, the model by Jaramillo et al. [17] predicts that even higher temperatures would result in shifts in the pest's latitudinal and altitudinal range. Yet, it seems that this erstwhile worse case scenario is already happening, as changes in the altitudinal range of *H. hampei* have recently been observed in Indonesia and Uganda; moreover, on the slopes of Mt. Kilimanjaro in Tanzania the coffee berry borer is now found at elevations 300 meters higher than those at which the insect was present ten years ago [24].

To the best of our knowledge, no information exists for predicted future distributions of the coffee berry borer under climate change scenarios for any coffee production areas in the world. In order to anticipate threats and prioritize management actions, we used the CLIMEX model [25], [26] in conjunction with HadCM3, a coupled atmosphere-ocean general circulation model, to assess the future distribution of *H. hampei* in the major *C. arabica* production zones of East Africa. The CLIMEX model relates present-day distributions to current climate and then projects the fitted climatic envelopes under future scenarios to identify how and where spatial shifts could occur [27]–[29].

## Results

**Figure 1:**
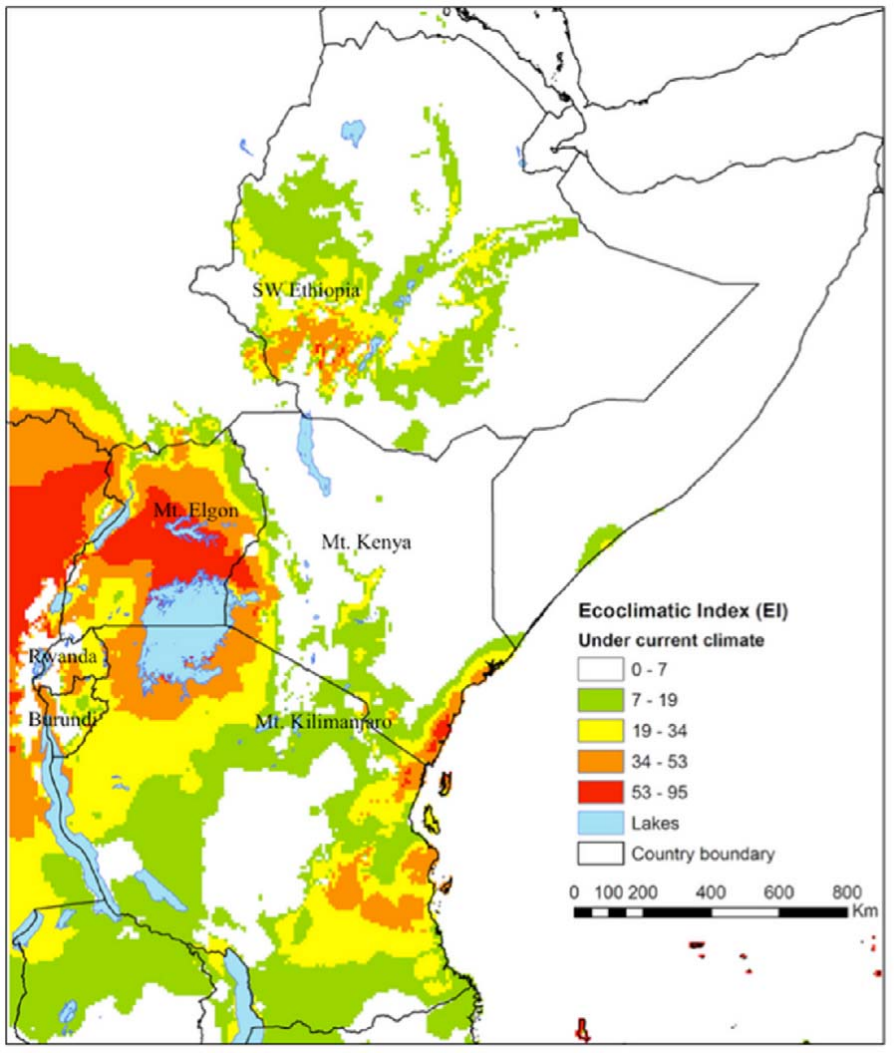
Distribution of the coffee berry borer (*Hypothenemus hampei*) in Eastern Africa under current climate, the map was constructed using the ecoclimatic indices (EI) obtained from CLIMEX parameters in **Table 1**. The EI values (0–100), indicates unsuitability of the location's climate (0), and a ‘perfect’ climate for the given species (100).

The CLIMEX parameters (Table 1) were inferred from field and laboratory data on the coffee berry borer bionomics [17], [30]–[31], or were estimated iteratively through manual adjustment until the model predictions produced a satisfactory match with the observed records. The values of the Ecoclimatic Index (EI) for current climate show that conditions are most favourable for *H. hampei* within the lowlands and some mid altitudes [900–1,800 meters above sea level (m.a.s.l.)] of Eastern Africa (Fig. 1). In this area, the coffee berry borer is currently particularly prevalent and damaging in the central and western regions of Kenya, throughout Uganda, southwestern Ethiopia, parts of southeast and southwest Rwanda and the entire eastern side of Mt. Kilimanjaro in Tanzania. The poor model fit in few localities is probably due to the quality and insufficiencies of on-the-ground available climate data.

**Table 1:** CLIMEX parameter values used for the coffee berry borer *Hypothenemus hampei* (Ferrari) predictive mapping*.

Parameter designation	Values†
**Temperature parameters**	
Lower threshold of temperature for populations growth (DVO)	14.9°C
Lower optimal temperature for population growth (DV1)	23°C
Upper optimal temperature for population growth (DV2)	30°C
Upper threshold temperature for population growth (DV3)	32°C
**Moisture parameters (proportion of soil moisture holding capacity)**	
Lower threshold of soil moisture (SM0)	0.25
Lower limit of optimal range of soil moisture (SM1)	0.7
Upper limit of optimal range of soil moisture (SM2)	1.2
Upper threshold of soil moisture (SM3)	2
**Cold stress indices**	
Temperature threshold of cold stress (TTCS)	0°C
Rate of accumulation of cold stress (THCS)	0 Week^−1^
Degree-days threshold of cold stress (DTCS)	32 d °C
Rate of accumulation of cold stress linked to degree-days (DHCS)	−0.0001 Week^−1^
**Heat stress indices**	
Threshold of heat stress (TTHS)	34.25°C
Rate of accumulation of heat stress (THHS)	0.002 Week^−1^
**Dry stress indices**	
Soil moisture dry stress (proportion of soil holding capacity) (SMDS)	0.2
Rate of accumulation of dry stress (HDS)	−0.015 Week^−1^
**Wet stress indices**	
Soil moisture wet stress (proportion of soild holding capacity) (SMWS)	2.67
Rate of accumulation of wet stress (HWS)	0.001 Week^−1^
**Annual heat sum indices**	
Degree-days threshold (PDD)	262°C

*Except for stress indexes values (see Materials and Methods section), all data used in this table is derived from real data on bionomics of *H. hampei* gathered in the field or in laboratory [19], [26], [27].

† Parameters without units are dimensionless.

**Figure 2:**
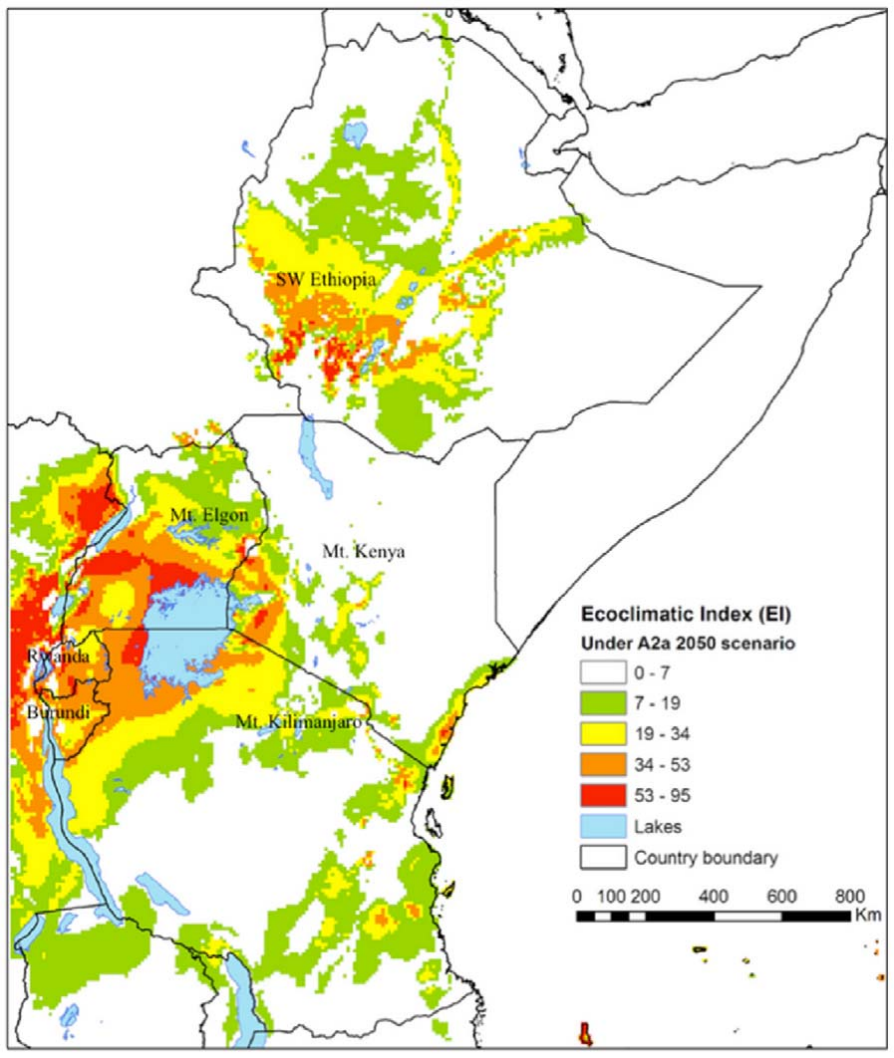
Climate suitability (EI) for the coffee berry borer (*Hypothenemus hampei*) in Eastern Africa under the climate conditions according to the HadCM3-SRES A2 scenario in 2050. The EI values (0–100), indicates unsuitability of the location's climate (0), and a ‘perfect’ climate for the given species (100).

Figures 2 and 4 show the climate suitability expressed as EI values for *H. hampei* in eastern Africa according to the HadCM3-SRES A2 and B2 climate scenarios (see below under *climate change scenarios*) in 2050. In both scenarios southwestern Ethiopia, the Ugandan part of the Lake Victoria and Mt. Elgon regions are predicted as highly suitable for the coffee berry borer, as well as the area around Mt. Kenya and the Kenyan side of Mt. Elgon, and most of Rwanda and Burundi (Figs. 2 and 4).

**Figure 3:**
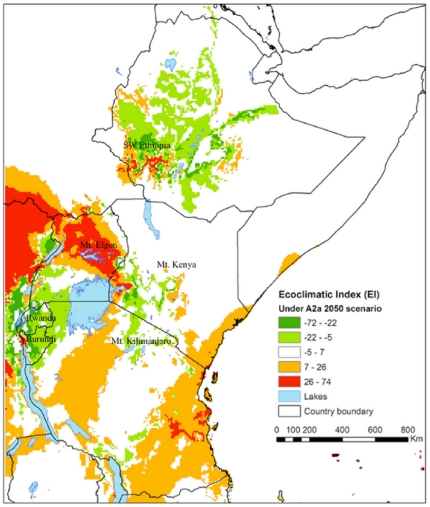
Distribution of the coffee berry borer (*Hypothenemus hampei*) illustrating species range shifts in Eastern Africa under climate change scenario A2A*. * The map was developed from the difference between the values EI for the predicted future *Hypothenemus hampei* distribution obtained when applying scenario A2A criteria (Figure 3) and the distribution under current climate in Eastern Africa (Figure 1).

Figures 3 and 5 present differences between the EI values of *H. hampei* for the A2A and B2A climate change scenarios and the current climatic conditions in eastern Africa. The objective was to identify future regions with either reduced or increased suitability for the coffee berry borer as well as cultivated *C. arabica*, as pest and host plant share, except for the optimum temperature, similar thermal tolerances [17].

**Figure 4:**
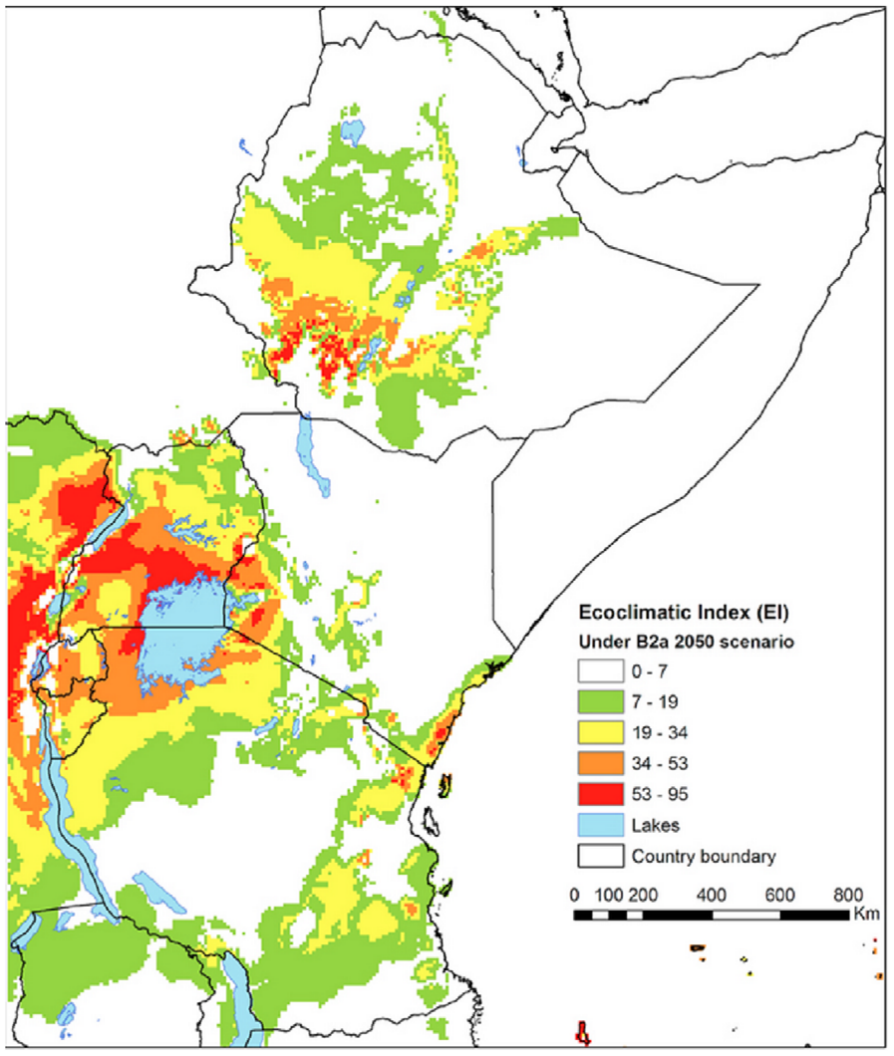
Climate suitability (EI) for the coffee berry borer (*Hypothenemus hampei*) in Eastern Africa under the climate conditions according to the HadCM3-SRES B2 scenario in 2050. The EI values (0–100), indicates unsuitability of the location's climate (0), and a ‘perfect’ climate for the given species (100).

**Figure 5:**
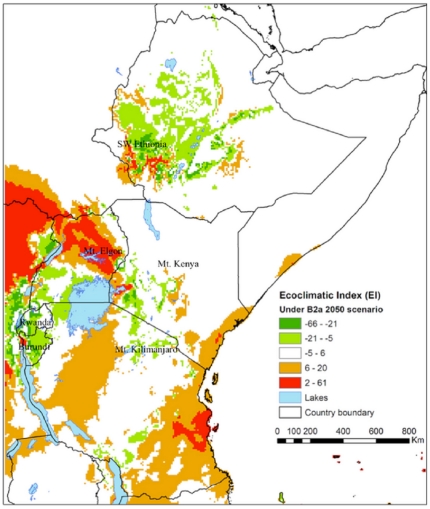
Distribution of the coffee berry borer (*Hypothenemus hampei*) illustrating species range shifts Eastern Africa under climate change scenario B2A*. * The map was developed from the difference between the values EI for the predicted future *Hypothenemus hampei* distribution obtained when applying scenario B2A criteria (Figure 4) and the distribution under current climate in Eastern Africa (Figure 1).

According to scenarios A2A and B2A (Figs. 3 and 5) the suitable area for coffee production will shrink in most of Kenya, Uganda, Rwanda and Burundi, whereas it will probably expand in Tanzania and Ethiopia. The prevalence of *H. hampei* is predicted to increase around Mt. Kenya, particularly in the coffee-producing areas of Embu and Meru, as well as in the western part of Kenya, around Kitale and Mt. Elgon.

Overall, the situation is forecasted to worsen in the current *C. arabica* producing areas of Uganda, particularly around the eastern side of Lake Victoria and Mt. Elgon. Likewise, the climatic suitability for coffee berry borer is predicted to increase in southwest Ethiopia, the most important core area for the natural distribution of *C. arabica* [16]. On the other hand, future conditions in Rwanda and Burundi are predicted to be less appropriate for *H. hampei* as suitable areas for *C. arabica* cultivation will decrease (Figs. 3 and 5).

For Mt. Elgon (Kenya and Uganda), Mt. Kenya (Kenya) and Mt. Kilimanjaro (Tanzania), the habitat suitability for the borer is forecasted to be low, indicating the possibility for altitudinal expansion of *C. arabica* cultivation in these areas (i.e., potential upslope movement of coffee plantations) (Figs. 3 and 5).

**Figure 6:**
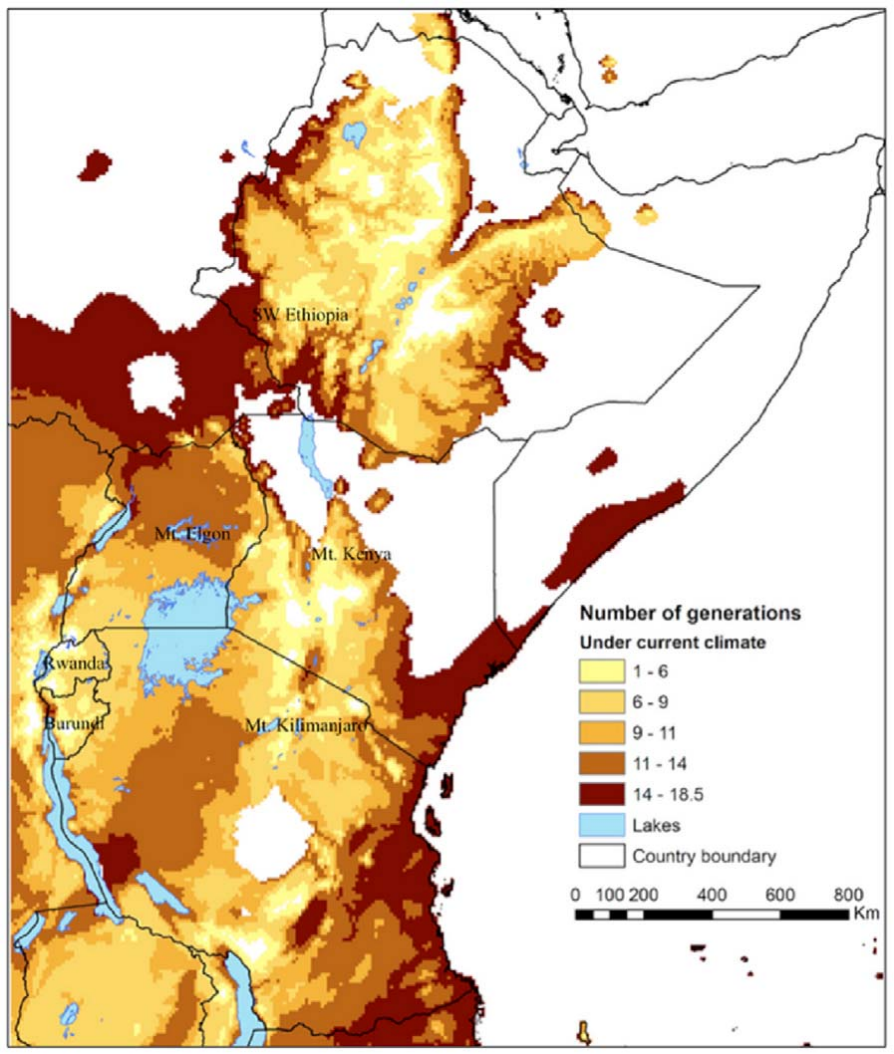
Spatial patterns in the number of coffee berry borer (*Hypothenemus hampei*) generations per year in Eastern Africa under current climate.

**Figure 7:**
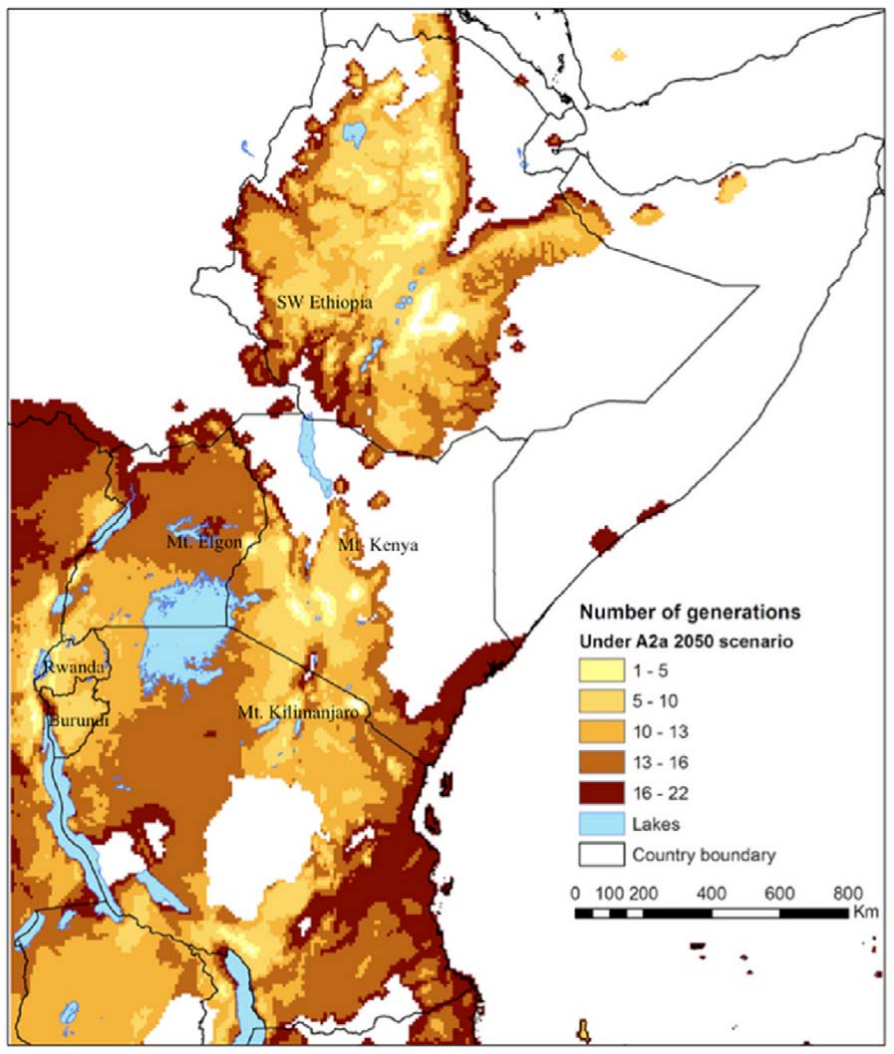
Spatial patterns in the number of coffee berry borer (*Hypothenemus hampei*) generations per year in Eastern Africa under the climate conditions according to the HadCM3-SRES A2 scenario in 2050.

**Figure 8:**
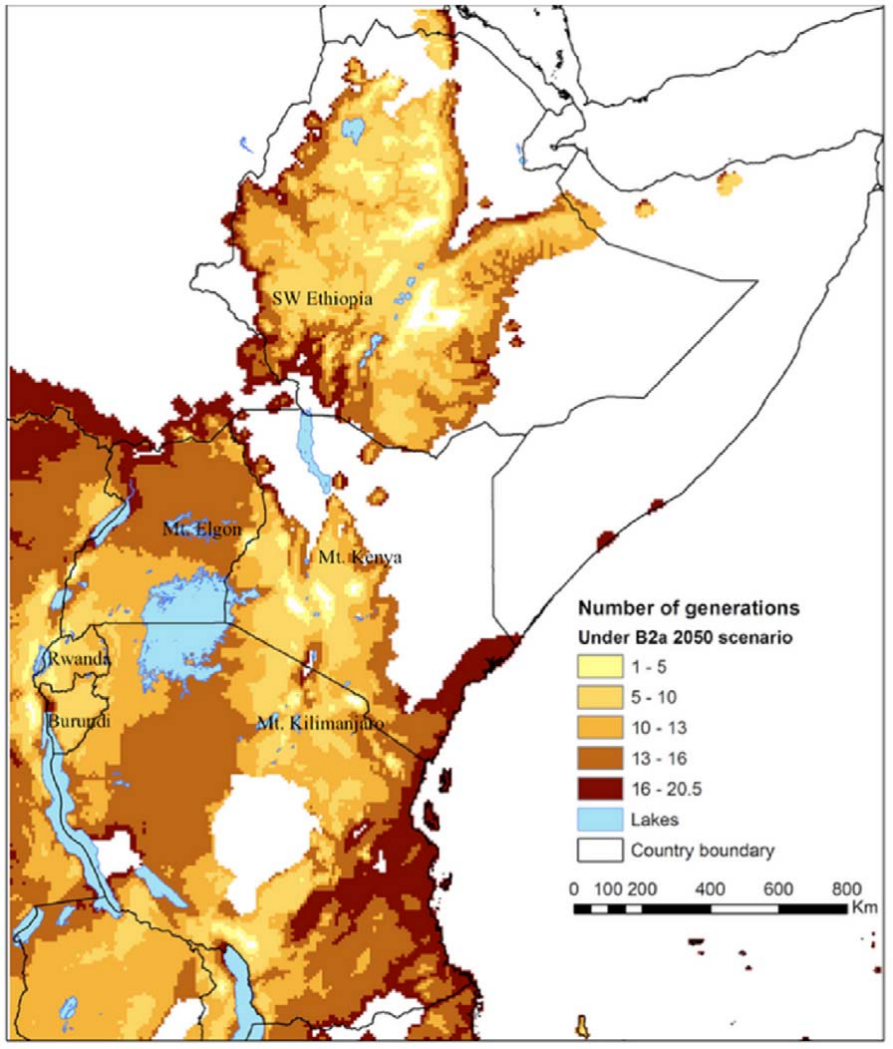
Spatial patterns in the number of coffee berry borer (*Hypothenemus hampei*) generations per year in Eastern Africa under the climate conditions according to the HadCM3-SRES B2 scenario in 2050.

CLIMEX estimates the number of generations of the insect solely based on the total number of degree-days above the lower temperature threshold for population growth. The predicted number of *H. hampei* generations per year range from ten in *C. canephora* growing areas in Uganda to two in the upper *C. arabica* areas in all East African countries under current climate conditions (Fig. 6). Changes in EI with climate translate into changes in generation time. The calculated hypothetical number of generation of *H. hampei* is predicted to increase in all middle altitude *C. arabica* producing areas (Figs. 7, 8). Whereas currently, the coffee berry borer is able to complete between 1–4.5 generations in East Africa [17], under both climate change scenarios used in this study, by 2050 the number of generations will have increased to 5–10 and 11–16 in high (1,400–1,800 m.a.s.l.) and low to middle elevation (900–1,300 m.a.s.l.) coffee production regions of East Africa, respectively.

**Figure 9:**
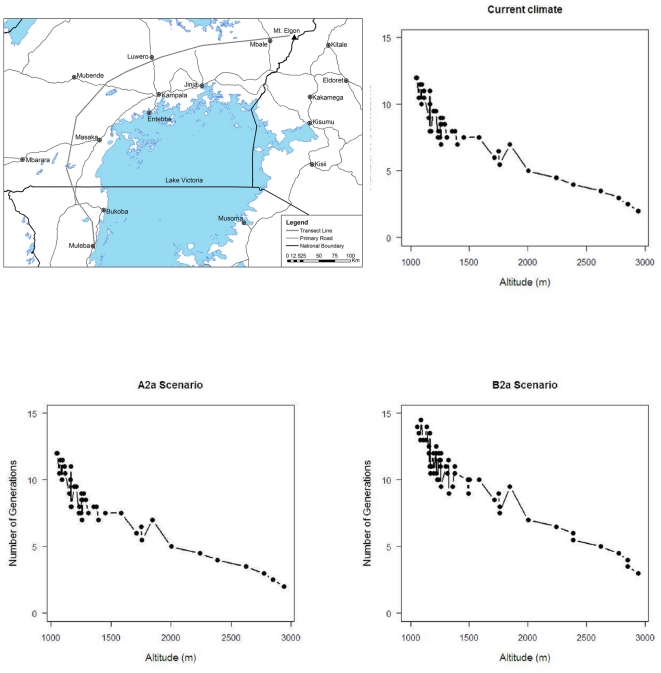
Number of coffee berry borer (*Hypothenemus hampei*) generations per year along an altitudinal transect around Lake Victoria (Tanzania, Uganda and Kenya) under current climate and according to the HadCM3-SRES A2 and B2 scenarios in 2050.

To better illustrate the effect of altitude on number of coffee berry borer generations, Figure 9 presents the predicted number of generations of *H. hampei* along an altitudinal gradient around Lake Victoria, which includes *C. canephora* plantations in Bukoba, Mubende, and -Luweero, and *C. arabica* cultivation areas in Mbale and the Mt. Elgon area. This clearly illustrates the changes in number of generations when moving upslope. For A2 case scenario, the number of *H. hampei* are not predicted to dramatically change compared to current climatic conditions, however, remarkable changes would take place under B2 scenario, where total number of generations of the borer would be around four even at altitudes close to 3,000 m.a.s.l.

## Discussion

Climate change is affecting the distribution, demography and life history of many species, particularly insects [1], [32], [33]. These changes are having, and will have, consequences for human livelihoods, including an increased spread of pest and diseases of important crops [32], especially in Africa, which is considered one of the most vulnerable continents to climate change and climate variability [1].

In 2009, Jaramillo et al. reported on the thermal tolerance of the coffee berry borer and its potential implications in a climate change environment [17]. Their model forecasted that a 1–2°C increase could lead to an increased number of generations, dispersion and damage by the coffee berry borer; whereas a rise in temperature of 2°C and above could lead to shifts in altitudinal and latitudinal distribution of the pest. Only two years later, there are strong indications that these changes are already occurring, with grave implications for the coffee industry (http://www.coffeeclubnetwork.com/redes/form/post?pub_id=2593). The coffee berry borer is already present in East Africa at altitudes >1,800 m.a.s.l [35] and recent reports from Tanzania indicate that the insect has moved up 300 m.a.s.l during the last ten years [24], [36]. In addition, the present La Niña event is causing unusually warm and dry conditions throughout East Africa, leading to serious outbreaks of *H. hampei* in the region, for example in Rwanda (Fabrice Pinard, CIRAD, pers. comm. 2011).

In this study we present predictions of future distributions of *H. hampei* in East Africa by 2050 under two climate change scenarios. The objective was to elucidate how such shifts may affect the region's *C. arabica* production in the future, in order to timely develop appropriate adaptation strategies. To the best of our knowledge this is the first report of its kind, as no studies on future distribution of the coffee berry borer exist for any coffee producing country or region across the globe. According to our predictive mapping, which is based on well-documented life history traits of *H. hampei* [17], by 2050 the coffee berry borer will be particularly damaging in current areas of high quality *C. arabica* coffee production in East Africa, in medium to higher altitudes ranging from 1,200 to 1,800 m.a.s.l., where *H. hampei* is likely to thrive in the future. According to Eitzinger [37] the current optimum elevation for *C. arabica* is 1,400–1,600 m.a.s.l., but this is forecast to shift to 1,600–1,800 m.a.s.l by 2050, due to raising temperatures. In the tropics, where the altitudinal temperature gradient is vastly steeper (>1000 times) than in the temperate zones, upslope range shifts are the most commonly expected response of species ‘escaping’ a warming climate [38]. Our study also predicts that the number of generations of *H. hampei* will increase along an altitudinal gradient as a response of raising temperature (Fig. 9). Thus, areas currently considered as marginally suitable for the borer will become favourable for population persistence in the future. The number of generations per fruiting season/year could increase throughout the region from the current 1–4.5 to 5–10, and some of our results even indicate up to 10–16 generations of the insect within a year/fruiting season in certain low to mid-altitude regions of East Africa (Figs. 7 and 8). Nevertheless, due to the limited carrying capacity of the coffee berries and predicted changes in rainfall patterns, more than ten generations of the insect per year seem unrealistic. Consequently in these areas of East Africa, *C. arabica* production most certainly will need to be moved to higher elevations. It has been estimated that Colombian *C. arabica* plantations would have to be moved by 167 m in altitude for every 1°C of increase in temperature, in order to maintain the same productivity and quality [39]. Although these figures cannot be directly extrapolated for East Africa, it gives an idea of the magnitude of a potential distribution shift. An assisted altitudinal migration of *C. arabica* coffee plantations in East Africa would most probably not be feasible, because of a paucity of available and suitable high altitude habitats in East Africa, and due to rising demographic pressure and issues related with food security that the region is likely to face in the future. Kenya, Uganda, Tanzania, Rwanda and Ethiopia are predicted to experience population increases of 77–110% by 2050 (Population Reference Bureau, http://www.prb.org). Moreover, climate change represents an immediate and unprecedented threat to agriculture in Africa. Climate change projections for the continent suggest that, by the end of the twenty-first century, climate change will have a substantial impact on agricultural production and consequently on the scope for reducing poverty [40]. Today, most of sub-Saharan Africa is still largely an agrarian economy, with this sector being overwhelmingly responsible for livelihood creation, food security and income generation [41]. The IPCC [1] forecasts a 10–20% decline in overall global crop yields by 2050, and even predicts that in some African countries yields from rain fed agriculture may fall by up to 50% by 2020. Additionally, across the continent, arid and semi-arid areas are expected to expand by up to 8% by 2080, corresponding to a reduction of approximately 60–90 million hectares of agriculturally productive land [42]. Thus, in such a scenario, it is not very likely that the ever-shrinking arable land in Africa would be used for crops like coffee, but rather to grow food crops. Furthermore, even if land at higher elevations is available, it is not clear whether soil factors would be adequate for coffee production.

The International Coffee Organization (ICO) predicts that under the A2 and B2 climate change scenarios, coffee production will decrease by up to 10% compared to the reference case without climate change [43]. According to the ICO, the highest yield reductions are expected in Africa and South America, with inherent consequences for coffee prices worldwide. Yet ICO's forecasts consider only abiotic stress (i.e., the impact of rising temperatures and changes in rainfall patterns on the physiology of the plants), whereas the model presented in this paper takes into consideration also a cosmopolitan and damaging pest of coffee – the coffee berry borer. Recent studies suggest that climate change will not only influence plant performance, but also its interactions with other trophic levels, consequently affecting the abundance of the species [44], [45]. For example, decoupling of the coffee berry borer and its natural enemies could result in higher pest numbers or more serious outbreaks. Presently, nothing is known about the effects of a warming climate on the natural enemies of the coffee berry borer, yet, higher trophic levels are often disproportionately affected by drivers like climate change and habitat modification, with specialist natural enemies (parasitoids) more hit than generalists (predators) [45]. Accordingly, it is crucial to add estimates of future distributions of natural enemies of coffee pests and diseases into existing and yet to be developed models to enable better planning by growers and the coffee industry.

Between 2009 and 2011, *C. arabica* prices have increased by 160% (http://www.coffeeclubnetwork.com/redes/form/post?pub_id=2533), mainly due to dramatically reduced production levels in East Africa and Latin America, particularly in Colombia, which have been attributed to a large extend to extreme weather events (La Niña) leading to severe outbreaks of pests and diseases [12]. According to the ICO, climatic variability is the main factor responsible for the present oscillations of coffee yields in the world [43].

Climate change and its forecasted impact on coffee production will have huge implications for livelihoods and poverty levels throughout the tropics. Most studies agree that climate change will cause more harm to poor communities [46] like small-scale coffee producers because they rely more heavily on natural resources for survival and have little capital to invest in costly adaptation strategies and/or pest and disease management. Seventy percent of the world's commercial coffee production is carried by often impoverished small-scale farmers, and in total 120 million people depend directly or indirectly on coffee for their subsistence [34]. These production systems are especially vulnerable to climate change because many of the famers solely grow coffee on their farms and consequently have to invest a significant share of their commodity revenues into purchasing food. Thus, climate change effects on coffee cascade into worsening food security, malnutrition and ultimately, poverty. In 2007 a survey conducted by the International Center for Tropical Agriculture (CIAT) with 179 small coffee farmers in Mexico, Nicaragua and Guatemala, revealed that over 67% of them and their families were unable to maintain their normal diet for 3–8 months of the year [47].

If we add to this picture pest and disease outbreaks, the farmers would have to use the income generated from coffee in plant protection strategies such as managing pests like *H. hampei*. In many respects, climate changes are likely to be more devastating for crop production if they lead to sudden pest outbreaks because control measures are difficult to apply quickly enough or on a sufficiently large scale to contain the problem [48], and even more so for poor subsistence farmers, like the majority of coffee growers. Climate change is expected to make coffee production more difficult and unpredictable, resulting in alternating periods of over- and underproduction. Hence, there is an urgent need to develop efficient and affordable adaptation strategies for coffee cultivation that include management of insect pests like the coffee berry borer, in a changing climate.

Possibly the best way to adapt production technologies to a rise of temperatures in coffee plantations is the introduction of shade trees, which alter the microclimate and create a diversified and therefore more resilient coffee agroecosystem that will perform better under climate change [49]–[51]. Positive effects of shade trees in coffee systems have been extensively demonstrated during the last years [e.g. 52]–[55]. Shade trees mitigate microclimatic extremes and can buffer coffee plants from microclimate variability [52], leading to a decrease in the temperature around the coffee berries by up to 4°C [56]. A reduction of 4°C would imply a drop of 34% in the intrinsic rate of increase of the coffee berry borer [17], therefore allowing to grow coffee in areas that will most likely experience increases in temperature and would be otherwise unsuitable for coffee production due to increased pest pressure. For example, shade levels of 40–60% provided by trees in Costa Rica helped maintain air and leaf temperatures below or close to 25°C [53]. Shade trees also play a role in soil and water conservation and management [52], which are critical issues, particularly in East Africa. Teodoro et al. [57] demonstrated that coffee berry borer densities were significantly lower in shaded versus unshaded coffee plantations, possibly because shade coffee agroecosystems can serve as a refuge for beneficial arthropods (native and introduced), leading to higher levels of biological control of *H. hampei* [58], [59]. Additional benefits of have been demonstrated in a two-year study of shaded and sun-grown coffee in the Kiambu area of Kenya: coffee berry borer infestation levels in the shaded plantation were always lower than the sun-grown coffee, and remained below the 5% economic threshold level, an effect most likely due to the lower temperatures in the shaded coffee plantations. Lower pest numbers were accompanied by considerably higher yields in shade compared to sun-grown coffee, possibly because of improved soil and nutrition conditions, and water management in the former, contradicting earlier reports of inferior yield performance of shade vis-à-vis sun-grown coffee (J. Jaramillo et al., unpublished data).

Boko *et al*. [42] noted that very little research has been done in Africa on the impacts of climate change on functional agro-biodiversity (including work on insect pests) and their interactions/impacts on crop production. Consequently, there is a critical need to address the problem of inadequate capacity for adaptation to climate change in Africa because of insufficient information and understanding on the status and trends in ecosystems. The aims of this study were to fill some of the climate change knowledge gaps in the coffee-production sector, and to assist in the development of an adaptation strategy package for climate change on coffee production. Small-scale coffee farmers, particularly in Africa, have little capital to invest in possible climate change adaptation strategies, lowering their resilience to changing conditions. Our predictive mapping of future coffee berry borer distribution and reproductive biology in East Africa clearly demonstrates the enormous impacts of climate change on the crop. We believe that the use of shade trees in the framework of more diversified coffee plantations (e.g. by introducing food crops to the system) to suppress coffee pests like the coffee berry borer is rational, affordable, and relatively easy for coffee farmers and other stakeholders to implement, constituting one of the many adaption strategies needed to improve the resilience of agricultural systems, especially in the tropics, in a changing climate. It will also provide essential ecosystem service benefits at the local and regional levels.

## Materials and Methods

### Occurrence data

A database of the distribution of *H. hampei* in Africa (presence data only) and specifically in the eastern part of the continent was created using data determined from field surveys (J. Jaramillo, unpublished data), scientific publications and reports [13], [14], [34]–[36], [60]–[63], and the internet-based search engine of the Global Biodiversity Information Facility (http://www.gbif.org). The total number of data points was 114.

### The model

Various modelling tools have been used for predicting species distributions according to regional climates [64]. The modelling program CLIMEX version 3.0 [65] was used to infer the climatic requirements of the coffee berry borer from its current distribution it its native range, and to project its potential distribution in Africa (create an ecological niche model of the pest), with input data on a monthly scale (minimum and maximum temperature, relative humidity at 9:00 am and at 3:00 pm and precipitation). The CLIMEX program is a flexible modelling and mapping tool used to create ecological niche models for, among others, insects, especially agricultural pests, which combines actual data on the bionomics of a given species and/or the observed distribution and abundance data of it to estimate its optimal climate and climate tolerance limits for modelling its potential future distribution [66]. CLIMEX integrates weekly responses of a population to moisture and temperature and calculates annual indices from these. There are two aspects to a species' response to these variables. CLIMEX uses a set of fitted growth and stress functions to assess the potential for a species to persist and grow at each location for which relevant climate data are available. The growth index (GI), represents the suitability of the location for growth and development, and is calculated according to how close ambient temperatures (soil moistures or day-lengths) are to a species' optimal preferences, and the stress indices (SI) which relate to how the stress factors, like prolonged periods of cold, wet, hot or dry weather or pair-wise combinations of these factors, limit the geographical distribution of the species.

These indices are calculated as follows:
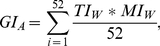
(1)

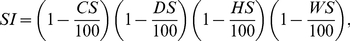
(2)

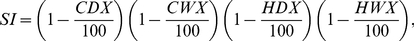
(3)

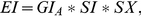
(4)Where: TIW and MIw are the weekly temperature and moisture, respectively, 52 is the number of weeks in a year. CS, DS, HS and WS are the annual cold, dry, heat and wet stress indices, respectively. CDX, CWX, HDX and HWX are the annual cold-dry, cold-wet, hot-dry and hot-wet stress interactions indices, respectively.

After fitting parameters for a particular species using either the built-in or supplementary weather station data, CLIMEX calculates growth (as a function of temperature, diapause, light and moisture) and stress (heat, cold, dry and wet) indices to indicate the suitability of the climate for each location. Growth and stress indices are then combined to generate the Ecoclimatic Index (EI), which indicates how favourable each location may be for that particular species. The EI values are in the range 0–100, where 0 indicates unsuitability of the location's climate, and 100 denoting a ‘perfect’ climate for the given species [65].

The ecological niche model for *H. hampei* was developed using actual data on the bionomics and life history traits of the coffee berry borer derived from published data on laboratory and field studies [17], [30], [31] (Table 1). The soil moisture index and the wet and dry stresses were adjusted so that the most favourable climate coincided in areas with a relative humidity of approximately 50–80%. The light, diapause, cold–dry, cold–wet, hot–dry and hot–wet stress indices were not used. Model parameterization was conducted for Ethiopia, Kenya, Uganda, Rwanda, Burundi, and Tanzania. The remaining African countries (Angola, Benin, Cameroon, Central African Republic, Chad, Congo, Equatorial Guinea, Gabon, Ghana, Guinea, Ivory Coast, Liberia, Malawi, Mozambique, Nigeria, Sao Tome & Principe, Senegal, Sierra Leone, Sudan, Togo, Zaire, and Zimbabwe) were treated as an independent data set and used for model validation. Once the African distribution of *H. hampei* was defined, based on a visual comparison of model output with observed distribution, EI values were compared to reported data on relative abundance. Published results related to abundance were used to refine parameter values so that highest EI values occurred where *H. hampei* was known to cause damage and lower values occurred where the species was less prevalent.

### Model validation

The model was validated by comparing output to reported distribution records in other parts of the world (data not shown). The model was applied to predict the population distribution of *H. hampei* in coffee growing countries of Asia (India, Sri Lanka, Vietnam, Thailand, Indonesia, Malaysia, and Philippines), Central America and the Caribbean (Guatemala, Honduras, El Salvador, Nicaragua, Mexico, Jamaica, Cuba, Dominican Republic, Costa Rica, and Haiti) and South America (Brazil, Colombia, Ecuador, Peru, Surinam, and Venezuela). Model outputs for these regions were compared to published coffee berry borer data [13], [14], [36], [67].

### Meteorological databases

The CLIMEX software is equipped with two climate databases, meteorological dataset and regular gridded dataset. The CLIMEX standard meteorological dataset consists of 30-year averages from 1961 to 1990 for an irregularly spaced set of around 2500 climate stations. Only 720 stations covered the African continent. Due to low density of weather stations in coffee growing areas in eastern African highlands, supplementary weather station data were extracted from the FAOCLIM database [68]. The climate variables required for CLIMEX included minimum temperature (*T_min_*), maximum temperature (*T_max_*), precipitation, and relative humidity (*RH%*). When unavailable the relative humidity was derived using the following formula:
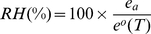
(5)In accordance with Allen et al., [69] this equation represents the ratio of the actual vapour pressure (*e_a_*) to the saturation vapour pressure *e°(T)* at the same temperature (*T*). The dewpoint temperature (*T_dew_*) was estimated using backward equations (eq. 5–10) when vapour pressure (*Ps*) is known. Then *T_dew_* was used in the calculation of *e_a_* and *T_max_* was used as the temperature in *e°*. The ecological niche model requires *RH%* at 09:00 and 15:00 hours. The *RH%* calculated using (eq. 5) was used as the *RH%* at 15:00 hours, and the *RH%* at 09:00 hours was calculated by dividing *RH%* at 15:00 hours by 0.85.
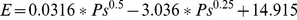
(6)

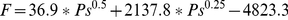
(7)

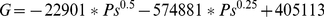
(8)

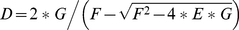
(9)

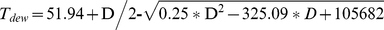
(10)


### Climate change scenarios

The climate change scenarios for 2050 presented in this paper are based on the Hadley Centre for Climate Prediction and Research's General Circulation Model (HadCM3) [62], one of the global circulation models presented by the IPCC's Third Assessment Report. The HadCM3 model was chosen because it provides good median results for Africa compared with other models. The downscaled (5 arc-minutes spatial resolution) outputs of the model were obtained from www.worldclim.org. The simulations were run at the SRES A2 and B2 emissions scenarios. The A2 scenario assumes that population growth does not slow down and reaches 15 billion by 2100 [1], with an associated increase in emissions and implications for climate change. The B2 scenario assumes a slower population growth (10.4 billion by 2100) and precautionary environmental practices are implemented [1], yielding more conservative predictions of anthropogenic emissions.

We generated a regular gridded dataset of climate normals for the current conditions (1950–2000) from data available at www.worldclim.org to fine-tune the parameter fit. The climate normals dataset consisted of 62,803 points spaced on an approximately 10×10 km regular grid for the Eastern Africa. Despite the slight rise in global temperatures since 1990, this should still provide the best indication of the current risk. An R script was used to transform the data format and estimate the values for relative humidity variables needed in CLIMEX. The *T_dew_* temperature was estimated using *T_min_* in the calculation of *e_a_* and *T_max_* was used as the temperature in *e°* (eq. 5). This estimate may not be accurate for arid areas [69], but because our study focused exclusively on non-arid regions of Eastern Africa, we considered that (eq. 5) provided a reasonable estimate of *RH%*. The *RH%* calculated using (eq. 5) was used as the *RH%* at 15:00 hours, and the *RH%* at 09:00 hours was calculated by dividing *RH%* at 15:00 hours by 0.85.

## Supporting Information

Abstract S1 Spanish Abstract S1.
(DOCX)
