# Supplementary material for: Some Like It Hot: The Influence and Implications of Climate Change on Coffee Berry Borer (Hypothenemus hampei) and Coffee Production in East Africa
Source: PLoS One. 2011 Sep 14;6(9):e24528. doi: 10.1371/journal.pone.0024528 (PMC3173381; doi:10.1371/journal.pone.0024528)
Supplement: Abstract S1 — Spanish Abstract S1. (DOCX) [file pone.0024528.s001.docx]

**S1**

**RESUMEN**

Los efectos negativos del cambio climático ya son evidentes para muchos de los 25 millones de caficultores en el trópico y para la industria del café, cuyo valor anual se calcula en 90 billones de dólares. La broca del café (*Hypothenemus hampei*), la plaga más importante de café en todo el mundo, ya se ha beneficiado del aumento de la temperatura en el África oriental, lo cual se manifiesta en un aumento en el daño a los cultivos de café y la expansión de su área de distribución. Existe una necesidad imperiosa de mitigar el problema de la insuficiente capacidad de adaptación al cambio climático en África, debido a la escasa información y comprensión sobre la situación y tendencias de los ecosistemas. Con el fin de anticiparse a las amenazas y priorizar las estrategias de manejo de *H. hampei*, se presentan aquí por primera vez, mapas de la distribución futura de la broca del café en zonas productoras de café de África Oriental. Utilizando el modelo CLIMEX se relacionan distribuciones actuales del insecto y, a continuación se proyectan la situación en función de futuros escenarios (A2A y B2B) para el modelo de cambio climático HADCM3. En ambos escenarios, la situación de la plaga se prevé que empeore en la actual zona productora de *C. arabica* del sudoeste de Etiopía, la parte Ugandesa del Lago Victoria y la región del Monte Elgon. Así mismo en la región del monte Kenia, y la mayoría de Rwanda y Burundi. El calculado hipotético número de generaciones de *H. hampei* por año se prevé que aumente en todas las áreas productoras *C. arabica*, de cinco a diez. Se sugiere que la mejor manera de adaptarse a un aumento de las temperaturas en las plantaciones de café, podría ser a través de la introducción de árboles de sombrío en las plantaciones. Los objetivos de este estudio son llenar algunos de los vacíos en el conocimiento existentes en la industria del café, y la elaboración de un esquema para el desarrollo de un paquete de adaptación al cambio climático en la producción de café.
